# Twin Pregnancy in the Martina Franca Donkey Breed Managed by Natural Reduction and Post-Fixation Manual Crushing

**DOI:** 10.3390/ani14172512

**Published:** 2024-08-29

**Authors:** Maria Cristina Veronesi, Ippolito De Amicis, Brunella Anna Giangaspero, Jasmine Fusi, Domenico Robbe, Francesco Castelli, Augusto Carluccio

**Affiliations:** 1 Department of Veterinary Medicine and Animal Sciences, Università degli Studi di Milano, 26900 Lodi, Italy; maria.veronesi@unimi.it; 2 Department of Veterinary Medicine, University of Teramo, 64100 Teramo, Italy; ideamicis@unite.it (I.D.A.); bagiangaspero@unite.it (B.A.G.); drobbe@unite.it (D.R.); fcastelli@unite.it (F.C.); acarluccio@unite.it (A.C.)

**Keywords:** donkey, twin pregnancy, natural reduction, post-fixation manual crushing

## Abstract

**Simple Summary:**

In Equids, twin pregnancy is an unwanted event, and the traditional management of twin pregnancies involves the early, pre-fixation, manual crushing of one embryo before the 16th day after ovulation when the two embryos are still mobile. However, due to the high percentage of natural reduction of one embryo in unilaterally fixed twins, early post-fixation manual crushing management can also be proposed, allowing the time for natural reduction occurrence. The present study aimed to report data about managing twin pregnancies through natural reduction and post-fixation manual crushing in the Martina Franca donkey breed. The natural reduction of one embryo occurred at 20–21 days after ovulation in 87.5% of unilateral twin pregnancies, with 12.5% of cases requiring post-fixation manual crushing, and none of the bilateral twin pregnancies requiring manual crushing. The single embryo pregnancy rate at 28 days after ovulation was 93.8% for unilateral twin pregnancies and 88.9% for bilateral twin pregnancies, with an overall pregnancy rate of 92%. The live foal rate was 87%. Taken together, the results showed that waiting for the natural reduction of one embryo and using post-fixation manual crushing can be a practical option for the management of twin pregnancy in donkeys.

**Abstract:**

In Equids, undetected twin pregnancy represents the most important cause of abortion and is also associated with high neonatal mortality rates. Therefore, the detection and management of twin pregnancies is pivotal to allow the continuation of single pregnancies. Although pre-fixation manual crushing of one embryo is the most common management of twin pregnancies, and the impact of natural reduction has been reported in mares, very little is known about donkeys. The present study aimed to report results on the natural reduction occurrence and post-fixation manual crushing management of twin pregnancies in the Martina Franca donkey breed. Methods: Twenty-five twin pregnancies were detected at 11–13 days after ovulation. At 16 days after ovulation, twin pregnancies were classified as unilateral or bilateral and left untreated. The occurrence of natural reduction of one embryo was assessed at 20–21 days after ovulation, and post-fixation manual crushing of one embryo was performed in those cases in which natural reduction did not occur. The pregnancy rate at 28 days after ovulation and live foal rate were recorded. Result: Sixteen out of 25 twin pregnancies were unilateral and nine bilateral. At 20–21 days after ovulation, the natural reduction of one embryo occurred in 87.5% of the unilateral and in none of the bilateral twin pregnancies. The remaining twin pregnancies were treated by post-fixation manual crushing of one embryo. The 28-days-after-ovulation single embryo pregnancy rate was 93.8% for unilateral twin pregnancies and 88.9% for bilateral twin pregnancies, with an overall pregnancy rate of 92%. The live foal rate was 87%. Conclusions: The success rate of natural reduction within 20–21 days after ovulation, the 28-days-after-ovulation pregnancy rate, and the live foal rate suggest that waiting for the natural reduction of one embryo and choosing the post-fixation manual crushing of one embryo could be considered as an alternative to traditional early pre-fixation manual crushing in the Martina Franca donkey breed.

## 1. Introduction

In Equids, twin pregnancy represents the most important cause of abortion and is also associated with high neonatal mortality rates [1,2,3,4,5,6], although the routine use of early ultrasound pregnancy diagnosis has strongly reduced its impact. Due to the characteristics of the Equid placenta, twin pregnancies are difficult to maintain and considered high-risk pregnancies, with a successful pregnancy rate of 10% reported in horses [7], which can compromise reproductive career [8]. Twin pregnancy rates in horses vary within the population and depend on the breed, age, reproductive status of the mare, and season, and a hereditary predisposition is also considered a factor [9]. The prevalence of twin pregnancies in the equine species varies from 1% to 15% [7,10,11,12,13,14,15]. The establishment of a twin pregnancy is due to the prolonged ovulatory peak of luteinizing hormone (LH), capable of inducing multiple ovulations, resulting in the fertilization of two different oocytes and the development of two dizygotic twins. Early transrectal ultrasound diagnosis of pregnancy can promptly identify a twin pregnancy, allowing timely intervention to prevent negative outcomes linked to this condition and drastically reducing the abortion rates associated with the continuation of the pregnancy [5,6]. An early transrectal ultrasound examination, between the 12th and 14th day after ovulation, allows an initial detection of the two vesicles in the uterus, which, similarly to a singleton pregnancy, migrate independently of each other approximately eleven times a day in the uterine cavity, ending with fixation at around the 16th day after ovulation [16]. In twin pregnancies, during the pre-fixation period, the percentage of early embryo natural reduction is irrelevant and comparable to that of single pregnancies [2]. The uterine fixation of embryonic vesicles around the 16th day after ovulation allows the classification of twin pregnancy, which, based on the location of the embryo, can be defined as unilateral when the two vesicles are in a single horn or bilateral when the two vesicles are distributed in the two uterine horns [2,4,17]. The different location of the two embryos influences the twin pregnancy management [18]. According to Ginther’s theory, indeed, the type of fixation of the two vesicles determines whether one, both, or none of the twins will survive [4]. In unilateral twin pregnancies, rates of early embryonic natural reduction are high and attributed to a nutritional deficit: indeed, the natural reduction of one of the two embryos occurs through the deprivation of the maternal embryo exchange [6]. This is achieved by a competitive absorption of nutrients linked to the size and position of the two vesicles in the early stages of pregnancy and, subsequently, to the orientation of the embryo within the vesicle [4]. This “deprivation theory”, proposed by Ginther (1989) [19], explains how the interference between the two embryonic vesicles during fixation causes a different orientation compared to that necessary to guarantee normal embryonic nourishment [2]. Orientation, in turn, is influenced by the embryonic relative size; vesicles with different sizes easily assume an orientation that provides a reduced contact surface, while embryos of similar sizes tend to reach equivalent endometrial contact areas. The embryo that survives natural reduction is not adversely affected during development, as abortion rates during the pre-fixation period are similar to singleton pregnancies [4]. Naturally reduced pregnancies in unilateral fixation can reach a success rate of 85% by 40 days after ovulation [19].

In bilateral fixations, the most frequent outcome is the abortion of both fetuses between the 40th and 90th day of pregnancy, while the rate of spontaneous embryo natural reduction up to 40 days of pregnancy is irrelevant [19].

The traditional management of twin pregnancies involves the pre-fixation manual crushing of one embryo before the 16th day after ovulation, when the two embryos are still mobile and can be easily separated, to crush the smallest one or the ones needing the least amount of uterine manipulation [20]. However, due to the high percentage of natural reduction in unilaterally fixed twins [19], early post-fixation management, allowing the time for natural reduction occurrence, can be also proposed [18]. 

Many donkey breeds, among them the Martina Franca breed, are currently considered endangered and need species-specific reproductive knowledge for population improvement programs. To the authors’ knowledge, some aspects of twin pregnancy in donkeys have been little or never investigated. Apart from studies about a genetic predisposition to twinning [9], the prevalence of double ovulations and the possible role played by the jenny’s age and reproductive status in twinning are lacking. Likely, the management of twin pregnancies and the outcome of managed twin pregnancies in terms of foaling rate, pregnancy length, and characteristics of the foals are not available. 

Therefore, the present study aimed to report retrospective data about the natural reduction and post-fixation manual crushing success rate in twin-pregnant Martina Franca donkeys.

## 2. Materials and Methods

### 2.1. General Information

This retrospective study was performed on a database belonging to the Veterinary Teaching Farm (VTF) of the Department of Veterinary Medicine, University of Teramo (Italy).

All the jennies were housed at the VTF (latitude of 42°43′34.351″ N; longitude of 13°46′21.539″ E, altitude of 270 m above sea level), where animals are kept under a natural photoperiod and the jennies are housed in small groups in outdoor paddocks. The jennies were fed with standard hay ad libitum and supplemented with 2 kg/jenny of commercial equine fodder. 

All the jennies underwent regular clinical examinations to assess their general health status and were submitted to regular deworming and vaccination protocols.

### 2.2. Reproductive Data

Data of 618 estrous cycles, collected from 212 Martina Franca adult females, recorded over 5 years (2019–2023), were considered. 

Age, body weight, and reproductive status of each jenny were recorded, considering as maiden those jennies never mated/inseminated, as barren those resulted not pregnant in the previous year, and as lactating the postpartum jennies with nursing foal. In the lactating jennies’ group, only data about animals with normal parturition and postpartum were considered. Moreover, according to VTF rules, none of the jennies were inseminated at the foal heat to avoid the increased risk of multiple ovulations, as reported by Carluccio et al. [21].

In all jennies, the management of estrus included the following schedule: about 12 h after detection of estrus signs, ovaries were monitored by transrectal ultrasonography with a portable ultrasound machine equipped with a 7.5–10 MHz linear probe (GE Logic Book^®^ XP; GE Healthcare, Little Chalfont, UK). Follicular growth was monitored every 12 h until the detection of a 35 mm diameter follicle, and then every 12 h until ovulation occurrence. Multiple ovulations were recorded and classified as unilateral or bilateral. Uterine conditions, such as tone, edema, and absence of anechoic content were also considered before insemination.

According to the VTF rules, jennies with multiple ovulations were inseminated only when the estrus occurred in late spring–summer.

On the detection of a follicle ≥ 35 mm in diameter, artificial insemination was performed every 48 h until ovulation occurrence, with fresh extended semen collected from jackasses of proven fertility, randomly chosen, assuring about 800 × 10^6^ progressively motile sperm in a 15 mL volume dose.

The early pregnancy diagnosis was performed 13–15 days after ovulation. When twin pregnancy was detected, no attempts at early embryo manual crushing were made, and a second diagnosis was performed at 16 days after ovulation when, according to embryo fixation site, twin pregnancies were classified as unilateral or bilateral. Day 16 after ovulation was chosen as the day of fixation, characterized by the abrupt cessation of mobility (fixation), in agreement with what reported for horses and donkeys by Ginther [22].

At the same time, because of the recognized importance of different vesicle diameters in the occurrence of twinning natural reduction [2], the diameter of each vesicle was measured to assess differences >4 or <4 mm.

A third diagnosis was performed at 20–21 days after ovulation when the occurrence of natural reduction was recorded. All the bilateral twin pregnancies in which natural reduction did not occur were submitted to manual crushing of one embryo [23,24]. In unilateral twins, thanks to the gentle massage of the uterine horn between the thumb, index finger, and middle finger of the hand, the embryo facing toward the apex of the uterine horn, and therefore with lower surface contact with the uterus, was pinched. No anti-inflammatory therapies were performed.

At 28 days after ovulation, all the singleton pregnancies and the managed twin pregnancies were confirmed, and normal features of the embryo and heartbeat were assessed [25]. Moreover, the live foal rate (jennies foaling live foals/28 days after ovulation pregnant jennies), the overall pregnancy loss (losses detected at 28 days after ovulation plus losses occurred from 28 days after ovulation to foaling) pregnancy length, foals’ sex, and foals’ birth weight were recorded.

### 2.3. Statistical Analysis

The t-Student test was used to compare the means of age, body weight, and reproductive status in the twin and in the singleton pregnancies. 

Fisher’s exact test was used to assess possible differences regarding pregnancy rate at 28 days after ovulation, foaling of live foals, and overall pregnancy losses in the managed twin and singleton pregnancies. 

The t-Student test was used to compare pregnancy length and birth weight of the donkey foals born from the managed twin pregnancies and those born from singleton pregnancies. The two-way ANOVA test was used to assess the possible effect of twin/singleton pregnancy and donkey foal sex on birth weight and pregnancy length. 

Significance was set for *p* < 0.05 (SPSS, vers. 29 for Windows).

## 3. Results

The descriptive clinical data about age, body weight, and reproductive status of the 212 Martina Franca jennies included in this study are reported in Table 1.

According to the recorded data, multiple ovulations included only double ovulations, never triple ones. Descriptive data about double ovulation prevalence in the 212 jennies and its unilateral or bilateral distribution are reported in Table 2.

A total of 179 pregnancies were obtained from the 212 Martina Franca jennies (84.4%), with an average estrous cycles/pregnancy of 1.51, and a pregnancy/estrous cycle of 62%. 

Twin pregnancies accounted for 25/179 (14%) and singleton pregnancies for 154/179 (86%).

Clinical data about age, body weight, and reproductive status of the 25 jennies with twin pregnancies and the 154 carrying a singleton pregnancy are reported in Table 3.

The description of the successful establishment of a singleton pregnancy, assessed at 28 days after ovulation, following natural reduction or post-fixation manual crushing in the 25 twin pregnancies, according to unilateral or bilateral embryo location, is reported in Table 4.

About twin vesicle differences in size, among 16 unilateral twin pregnancies, seven (43.8%) showed a difference >4 mm, while nine (56.2%) <4 mm. The spontaneous natural reduction of one embryo occurred in eight out of nine (88.9%) of the <4 mm different vesicles, and in six out of seven (85.7%) of those with a difference >4 mm.

The pregnancy rate at 28 days after ovulation, live foal rate, and the overall pregnancy losses in the managed twin and singleton pregnancies are reported in Table 5.

Pregnancy length and birth weight of the 20 donkey foals born from managed twin pregnancies (males N = 8, 40%; females N= 12, 60%) and of the 125 donkey foals born from spontaneous singleton pregnancies (males N = 53, 42.4%; females N= 72, 57.6%) are reported in Table 6.

## 4. Discussion

Data from the present study showed a 28.8% double ovulation rate in the studied population of 212 Martina Franca jennies, a result that falls within the wide 5.3–38% range reported for donkeys [9] and within the 8–31% reported for horses [10]. In donkeys, a higher percentage of double ovulations has been reported in large-sized donkeys [9], and the Martina Franca is one of the largest Italian donkey breeds [26]. In horses, double ovulations were reported to be more frequent in some breeds, with hypothesized more susceptibility in some familial lines [10], a hypothesis supported by a recent study investigating genes involved in the mechanism of donkey twinning [9].

According to the pregnancy rate, the overall 84.4% obtained in the present study is fully satisfactory when compared to the 78–80% reported for donkeys [27,28], the 74% reported for horses [15], and the 89.6% reported for mares carrying mules [29]. 

A twinning rate of 14% was observed, very similar to the 14.3% reported by Carluccio et al. [21] for Martina Franca jennies inseminated at the foal heat and within the 40% maximum rate reported for donkeys by Van Branden et al. [27]. Moreover, the present study’s twinning rate is very similar to the highest 15.4% rate reported for the Thoroughbred horse [12]. However, it should be considered that, in the present study, the twinning rate could have been affected by the decision to not inseminate jennies at the foal heat and in early spring, so the actual features of the twinning rate in the Martina Franca donkey breed should be better investigated. 

When looking at the clinical characteristics of jennies with twin or singleton pregnancies, the results showed that jennies developing twinning pregnancies were, on average, significantly older than those developing singleton pregnancies (9.5 vs. 7 years old), in agreement with data reported for the horse mare [30]. According to the reproductive status, in comparison to jennies with singleton pregnancies, no significant differences were found, although twin pregnancies were observed less frequently in maiden (8 vs. 12%) and more frequently in lactating jennies (44 vs. 39%). Compared with data reported for the horse, the results obtained in jennies agree with findings reported for maiden mares (10.9 vs. 18.1%) but are different for lactating mares (56.3 vs. 63.8%) and for barren mares, in which a significantly higher percentage of twinning was found (32.8 vs. 18.1%). Therefore, based on the results of the present study, the reproductive status of the jenny does not seem to be associated with susceptibility to twinning in the Martina Franca donkey breed.

According to the distribution in unilateral or bilateral twin pregnancies, a greater prevalence of unilateral than bilateral (64 vs. 36%) twin pregnancies was observed, in agreement with the 71% of unilateral pregnancies reported in the horse mare [2]. Therefore, as reported for the horse, also in donkeys, most twin pregnancies display a unilateral embryo fixation. Also in donkeys, as in the horse, the embryo is characterized by a first period of intra-uterine migration to allow the maternal recognition of pregnancy, followed by location at the base of the uterine horn and a final fixation around the 16th day after ovulation. Possibly, the mechanisms leading to the final localization of the two embryos in donkeys could be considered the same as shown for the horse. As a speculation, differently to the horse, for which a similar prevalence of unilateral double ovulations and unilateral twins’ localization were reported, in the present study, a higher prevalence of bilateral double ovulations and unilateral twin embryo fixation was observed. 

When the management of twin pregnancies is considered, a post-fixation (20–21 days after ovulation) protocol for manual crushing was chosen for bilateral twin pregnancies and for those unilateral pregnancies not reduced spontaneously. This approach was chosen considering its practical application in the field. In fact, in most cases, Martina Franca donkeys are reared under rural conditions in which veterinary examinations could be restricted in number, so that the need for post-fixation twin pregnancy management could be more acute.

In unilateral twin pregnancies, the 87.5% natural reduction of one embryo at 20–21 days after ovulation can be considered very satisfactory, with only two pregnancies (12.5%) requiring the post-fixation manual crushing of one embryo. This result is very interesting considering that, in the horse, in natural twin pregnancies with unilateral fixation, the reported natural reduction rate within 20 days was 58% [19]. Although it could be difficult to identify the exact reasons for this high natural reduction rate, a species-specific “natural selection” based on the nutritional depletion of one embryo can be supposed. The following successful singleton pregnancy rate was 93.75% because one pregnancy was lost after the manual crushing of one embryo. This success rate related to unilateral twin pregnancy management can be considered very satisfactory if compared with the 90% success rate reported in the horse after manual crushing performed between 17 and 20 days after ovulation [23,24]. 

When the difference in embryo vesicle size was considered, in unilateral twin pregnancies, spontaneous natural reduction of one embryo occurred in 85.7% of cases with difference in size >4 mm and in 88.9% of cases with difference <4 mm, with the lack of significant difference highlighting the absence of a higher probability of natural reduction for vesicles with differences >4 mm as reported in the horse (70% for difference in size <4 mm; 100% for difference in size >4 mm) [31]. 

All the bilateral twin pregnancies underwent manual crushing of one embryo at 20–21 days after ovulation, considering the negligible possibility of spontaneous embryo reduction reported in the bibliography [19]. The following successful singleton pregnancy rate was 88.9%, higher than the 75% reported in the horse within 30 days after ovulation [23,24]. The live foal rate of the managed twin pregnancies was 87%, a bit lower compared to 91.2% of the singleton pregnancies and to the 95.8–100% reported in jennies inseminated at the foal heat or the second heat [21].

Mean pregnancy length was significantly shorter in the managed twin pregnancies than singleton ones (359 vs. 369 days), however, falling within the range of normality reported for the breed (352–376 days [32], 365 ± 7.3 days [21], 333–395 days [33]) and very similar to the average 365 days reported for donkeys [9], while birth weight was not different between donkey foals born by managed twin and singleton pregnancies, and the results are very similar to those reported in a previous study on the Martina Franca breed [33].

The proportion of male and female foals was similar in the managed twin pregnancies (males = 40%, females = 60%), and singleton pregnancies (males = 42.4%, females = 57.6%), with the opposite distribution in comparison to what was reported by Carluccio et al. [33] for the same donkey breed (males = 57.04%, females = 42.96%).

## 5. Conclusions

In conclusion, the present study provides data about twin pregnancy in the Martina Franca donkey breed managed by natural reduction and post-fixation manual crushing. The twinning rate seems to be a bit higher than the average reported for horses, without a clear influence of the reproductive status on twinning occurrence, characterized by a prevalence of unilateral fixation. A high rate of the natural reduction of one embryo within 20–21 days after ovulation in unilateral twin pregnancy was observed. At 20–21 days after ovulation, the post-fixation manual crushing of bilateral twin pregnancies and of unilateral pregnancies that did not undergo manual reduction led to a satisfactory 28-days-after-ovulation pregnancy rate and live foal rate. Therefore, the results of the present study showed that waiting for the natural reduction of one embryo and the 20–21-days-after-ovulation manual crushing of one embryo could be considered as an alternative to traditional early pre-fixation manual crushing in the Martina Franca donkey breed or as an alternative to other post-fixation techniques [34,35].

## Figures and Tables

**Table 1:** Descriptive clinical data about age, body weight, and reproductive status of the 212 Martina Franca jennies included in this study.

Jennies (n.)	Age (Years) Mean ± SD (Min–Max)	Body Weight (kg) Mean ± SD (Min–Max)	Maiden n. (%)	Barren n. (%)	Lactating n. (%)
212	10.5 ± 5.8 (2–20)	343 ± 20 (250–405)	23 (10.7)	98 (46.5)	91 (42.8)

**Table 2:** Descriptive data about double ovulation prevalence in the 212 Martina Franca jennies included in this study and its unilateral or bilateral distribution.

Jennies (n.)	Estrous Cycles (n.)	Estrous Cycles/Jenny Mean (Min–Max)	Double Ovulations/ Estrous Cycles n. (%)	Unilateral n. (%)	Bilateral n. (%)
212	618	2.9 (1–8)	178/618 (28.8)	54/178 (30.3)	124/178 (69.7)

**Table 3:** Clinical data about jennies’ age, body weight, and reproductive status in the 25 twin and 154 singleton pregnancies.

	Age (Years) Mean ± SD (Min–Max)	Body Weight (kg) Mean ± SD (Min–Max)	Maiden n. (%)	Barren n. (%)	Lactating n. (%)
Twin pregnancy (n = 25)	9.8 ± 2.43 ^a^ (3–16)	349 ± 12.4 (318–375)	2 (8)	12 (48)	11 (44)
Singleton Pregnancy (n = 154)	7.0 ± 3.70 ^b^ (3–17)	351 ± 11.5 (295–380)	19 (12.3)	75 (48.7)	60 (39)

^a,b^ within column denotes significant differences for *p* < 0.05.

**Table 4:** Description of the successful establishment of a singleton pregnancy, assessed at 28 days after ovulation, after natural reduction or post-fixation manual crushing in the 25 twin pregnancies, according to embryo location.

	Pregnancy n. (%)	Natural Reduction n. (%)	Post-Fixation Manual Crushing n. (%)	Confirmed Singleton Pregnancy at Day 28 n. (%)
Unilateral	16/25 (64)	14/16 (87.5)	2/16 (12.5)	15 */16 (93.75)
Bilateral	9/25 (36)	0/9 (0)	9/9 (100)	8 */9 (88.9)

* one unilateral and one bilateral pregnancy were lost at the ultrasound examination performed at 28 days after ovulation.

**Table 5:** Pregnancy rate at 28 days after ovulation, live foal rate, and the overall pregnancy losses in managed twin and singleton pregnancies.

Pregnancies	Pregnancies at 28 Days after Ovulation n. (%)	Live Foals n. (%)	Overall Pregnancy Losses n. (%)
Managed twin (n = 25)	23/25 (92)	20/23 (87)	5/25 (20)
Singleton (n = 154)	137/154 (89)	125/137 (91.2)	29/154 (18.8)

**Table 6:** Pregnancy length and birth weight of the 20 donkey foals born from managed twin pregnancies and of the 125 donkey foals born from spontaneous singleton pregnancies.

	Pregnancy Length (d) Mean ± SD (Min–Max)	Birth Weight (kg) Mean ± SD (Min–Max)
Managed twin pregnancies (n = 20)	359 ± 9.71 ^a^ (342–381)	32.5 ± 4.12 (23–41)
Singleton pregnancies (n = 125)	369 ± 11.08 ^b^ (331–392)	31.7 ± 4.23 (21–41)

^a,b^ within column denotes significant differences for *p* < 0.001.

## Data Availability

This publication contains all data generated or analyzed during the study.
